# Language performance and brain volumes, asymmetry, and cortical thickness in children born extremely preterm

**DOI:** 10.1038/s41390-023-02871-0

**Published:** 2023-11-03

**Authors:** Hedvig Kvanta, Jenny Bolk, Lina Broström, Daniela Nosko, Lexuri Fernández de Gamarra-Oca, Nelly Padilla, Ulrika Ådén

**Affiliations:** 1 Department of Women’s and Children’s Health, Karolinska Institutet, Solna, Stockholm, Sweden; 2 Division of Clinical Epidemiology, Department of Medicine, Solna, Karolinska Institutet, Stockholm, Sweden; 3 Department of Clinical Science and Education, Karolinska Institutet, Stockholm, Sweden; 4 Sachs’ Children and Youth Hospital, Södersjukhuset, Stockholm, Sweden; 5 Department of Paediatrics, Örebro University Hospital, Örebro, Sweden; 6 Department of Psychology, Faculty of Health Sciences, University of Deusto, Bilbao, Bizkaia Spain

## Abstract

**Background:**

Children born preterm are more prone to have language difficulties. Few studies focus on children born extremely preterm (EPT) and the structural differences in language-related regions between these children and children born at term.

**Methods:**

Our study used T1-weighted magnetic resonance imaging (MRI) scans to calculate the brain volumetry, brain asymmetry, and cortical thickness of language-related regions in 50 children born EPT and 37 term-born controls at 10 years of age. The language abilities of 41 of the children born EPT and 29 term-born controls were then assessed at 12 years of age, using the Wechsler Intelligence Scale for Children, Fifth Edition and the Clinical Evaluations of Language Fundamentals, Fourth Edition. The differences between MRI parameters and their associations with language outcomes were compared in the two groups.

**Results:**

Brain volume and cortical thickness of language-related regions were reduced in children born EPT, but volumetric asymmetry was not different between children born EPT and at term. In children born EPT the brain volume was related to language outcomes, prior to adjustments for full-scale IQ.

**Conclusions:**

These findings expand our understanding of the structural correlates underlying impaired language performance in children born with EPT.

**Impact:**

- The article expands understanding of the structure-function relationship between magnetic resonance imaging measurements of language-related regions and language outcomes for children born extremely preterm beyond infancy.
- Most literature to date has focused on very preterm children, but the focus in this paper is on extreme prematurity and language outcomes.
- While the brain volume and cortical thickness of language-related regions were reduced in children born EPT only the volume, prior to adjustment for full-scale IQ, was associated with language outcomes.
- We found no differences in volumetric asymmetry between children born EPT and at term.

## Introduction

Children born extremely preterm (EPT), with gestational age (GA) < 27 weeks, have atypical neurodevelopment, which is affected by neonatal morbidity and their early ex-utero environment.^1^ Children born preterm, and children born EPT in particular, tend to be more prone to language function difficulties than term-born children.^1–3^ It is important to understand the underlying structural mechanisms because language has been shown to be critical for the cognitive development of children, their academic achievements, and their well-being as adults.^2^

Canonical language organization is centered in two left areas of the brain and their homologs on the right side. The first is in Broca’s area, which is the left triangular and opercular part of the inferior frontal gyrus that has traditionally been known to be vital for expressive language. The second is in Wernicke’s area, in the left posterior superior temporal gyrus, which is important for receptive language.^4,5^

Our knowledge of language organization has advanced and numerous brain regions are now known to subserve language processing.^2^ Language is initially perceived in the primary auditory cortex, Heschl’s gyrus, and the secondary auditory cortex in the superior temporal gyri. After that, processing occurs in the ventral pathway, which is important for language interpretation, including the superior, middle, and inferior temporal gyri and the inferior parietal gyri (angular and supramarginal gyri). Then, the dorsal pathway plays a dominant role in language production, including the inferior frontal gyri (opercular and triangular) and supplementary motor area.^5–8^

A systematic review examined structural magnetic resonance imaging (MRI) measurements, including brain volumetry, and associations with language outcomes in children aged 6-19 born very preterm, defined as less than 32 weeks GA.^2^ The authors concluded that the results of the studies were inconsistent, mainly because they used diverse methods and studied different GAs and morbidities.^2^ Other studies have shown that decreased total gray matter (GM), total white matter (WM), and intracranial volume (ICV) were associated with language outcomes in children born very preterm.^9,10^ We are not aware of any previous studies that have investigated the volume of specific language-related regions and their association with language outcomes when children born EPT are school-age.

Left-right asymmetry of the brain is an important aspect of brain anatomy and alterations have been reported in children with neurobiological disorders and disabilities, such as dyslexia and autism spectrum disorder (ASD).^5,11^ Brain asymmetry can be investigated using different MRI modalities, including structural 3D MRI, which measures volumetric asymmetry indexes (AIs). The view that language is a left-lateralized function has been regarded as problematic over the years, as the right hemisphere plays an important role in language processing, even in mature adults.^11,12^ Previous studies have shown increasing brain asymmetry throughout childhood and up to 30 years of age.^13^ However, asymmetry is present as early as in infancy.^5,14^

We do not know how the volumetric asymmetry of the regions known to be important for language processing is affected by extreme prematurity after infancy.

Cortical thickness represents the radial columns of the cortex.^15^ Most evidence to date has demonstrated reduced cortical thickness in the language-related regions of children born preterm, compared to term-born controls during childhood and adolescence.^16–18^ One study reported no association between language assessment results and cortical thickness when children born very preterm children reached 18-21 years.^17^ Meanwhile, another study that examined 15 children born EPT found positive associations between their cortical thickness and language performance at 4-6 years of age, but only after the findings were adjusted for ICV.^16^ More studies are needed to investigate associations between cortical thickness and language outcomes for children born EPT, in order to expand our understanding.

Since language development in relation to prematurity has been mainly studied in very preterm populations, children born EPT warrant more attention.^2^ They spend one to two months more than very preterm children outside the womb during a critical window for neurodevelopment when there is an increase in brain volumes, synaptic density, subplate thickness, and glia proliferation.^19–21^ These processes are prominent in language-related areas in the temporal and frontoparietal regions.^19–23^ Thus, alterations in brain volumes and cortical thickness in these regions could be different in children born EPT than in children born very preterm, with possible associations to adverse language development. Previous studies have found indices of EPT birth leading to brain alterations that are similar, but more severe than those found in children born very preterm.^23^ But there are also more complex differences, including volumetric increases found adjacent to the decreases in children born EPT.^22,23^ Brain adaptation in the face of an early developmental risk can result in reorganization of the brain, why EPT birth can lead to other brain alterations than those following very preterm birth that should be studied exclusively.^24^

There is a known increased risk of neurodevelopmental disorders in children born EPT, and these children face an increased risk of ASD ranging from 8 to 10% in different studies, compared to between 1 and 2% in the general population.^25,26^ Our group has previously reported that ASD is associated with brain volume growth trajectories and brain asymmetry in children born EPT at term age, with an impact on language performance.^5,27^

This study aimed to compare children born EPT and at term in three ways. The first aim was to investigate differences in brain volumes, volumetric AIs, and cortical thickness in pre-defined language-related regions at 10 years of age. Secondly, we wanted to investigate any differences in the results of language assessments at 12 years of age. Finally, we aimed to investigate associations between language outcomes and brain volume, volumetric AI, and cortical thickness in the language-related regions of both groups.

## Materials and methods

### Study population and study design

This was a prospective, observational, population-based cohort study of children born EPT between 22 weeks and 0 days and 26 weeks and 6 days of gestation. It partly overlapped with a National Swedish Study that focused on the survival and outcomes of children who were born EPT and received active perinatal care.^28^

**Fig. 1:**
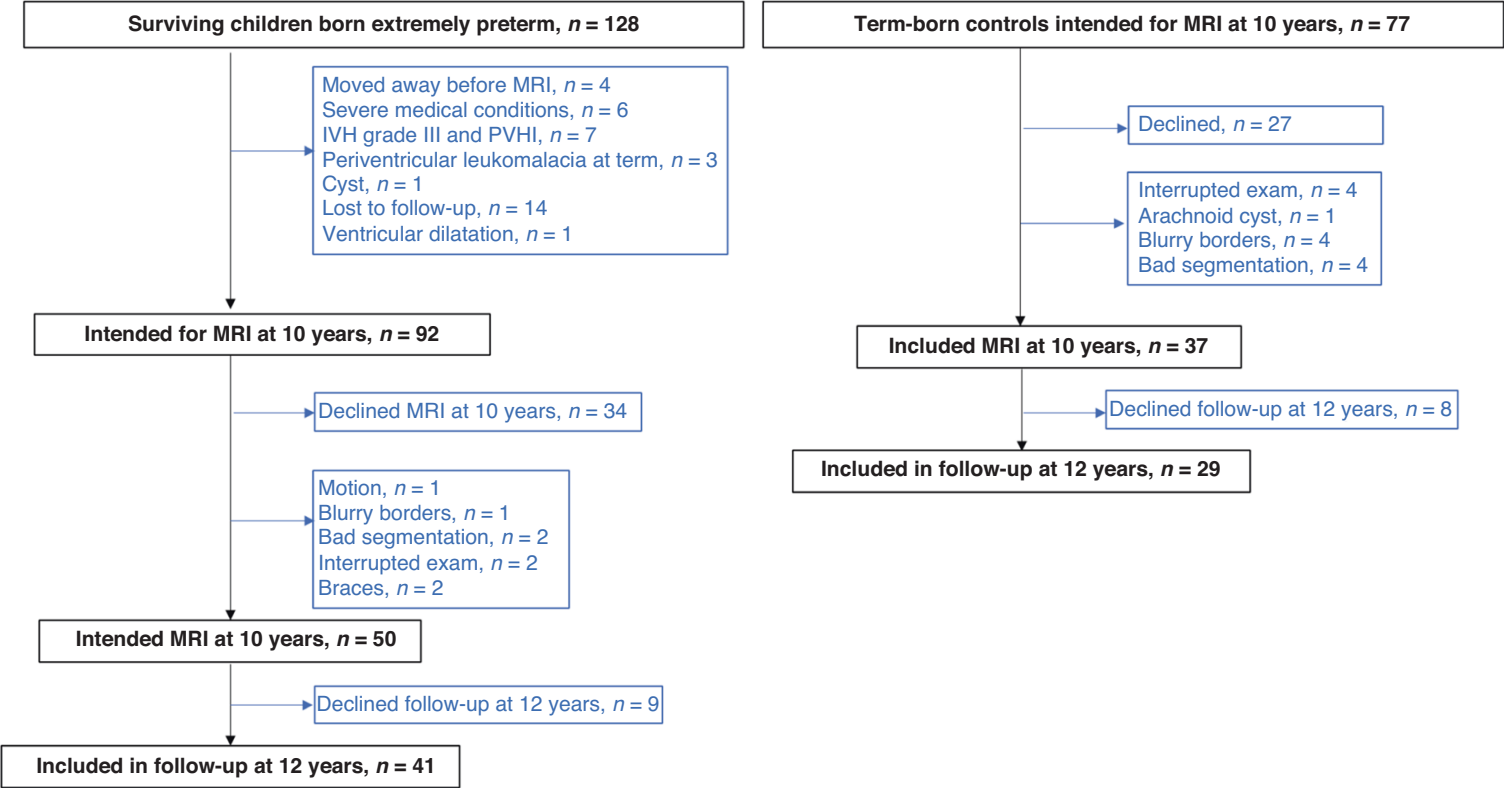
Flowchart of children born EPT and term-born controls. Summary of reasons for exclusion of children born EPT and term-born controls.

The study comprised 128 children who were born EPT in Stockholm from January 1, 2004 to March 31, 2007 and were still alive at term age, defined as 40 weeks and 0 days of gestation (Fig. 1). We excluded children with severe medical conditions, such as congenital malformations and chromosomal abnormalities. They were also excluded if they had brain lesions, namely cystic periventricular leukomalacia, intraventricular hemorrhage (IVH) grade 3, and periventricular hemorrhagic infarctions diagnosed with cranial ultrasound.^29^ The exclusion criteria also included focal brain lesions, cysts, and severe WM abnormalities on their MRI scans, as defined by a previously published scoring system.^30^ Those with low-quality MRI scans were excluded, defined as motion artifacts or blurring of the GM and WM interfaces.

The children born EPT were invited for a developmental assessment at 12 years of age.

Drop-out analyses were performed for the children who were included and those that were not included, because they did not meet the criteria, or their families declined to take part (Supplementary Tables 1 and 2).

The controls were singleton, term-born healthy children who were identified from the Swedish Medical Birth Registry at 2.5 years of age. They were matched to the children born EPT by place of birth, sex, day of birth, and maternal country of birth.^28^ We invited 77 of the Stockholm-born controls to undergo an MRI scan at around 10 years of age (Fig. 1) and they were also invited to a developmental assessment at 12 years of age. The global brain volumes of the same cohort have previously been assessed and published.^31^ We excluded one additional child-born EPT and one term-born control, due to insufficient quality of the segmentation.^31^

### Ethics

The ethics review board in Stockholm approved the study in accordance with the Helsinki Declaration. Written, informed consent was obtained from the parents.

### Baseline characteristics

Medical records were used to obtain perinatal data. Sepsis was defined as a positive blood culture or clinical symptoms of sepsis and an elevated C-reactive protein or leukocyte count. Small for gestational age was defined as a weight at birth of less than 2 standard deviations below the mean. Necrotizing enterocolitis was defined using Bell criteria.^32^ Bronchopulmonary dysplasia refers to the need for supplementary oxygen at 36 weeks of gestation. Patent ductus arteriosus (PDA) was defined as the need for either PDA ligation or treatment with ibuprofen. The clinical diagnosis of ASD was based on the diagnostic criteria in the Diagnostic and Statistical Manual of Mental Disorder 4th edition, and/or the International Classification of Diseases 10th revision, and the number of children born EPT in whom the diagnosis was made were verified at the time of MRI.^5^ At the time of the 12-year-old follow-up the parents were asked what language was used in the child’s home and this variable was dichotomized as everyone spoke Swedish in the household or at least one other language than Swedish was spoken in the household. Maternal education was dichotomized as mothers who did or did not attend university. Parents were asked which hand the children preferred to use or whether they used both hands equally at 6.5 years of age as part of the national follow-up.

### Language assessments

The children’s language function was assessed at 12 years of age. Both groups were examined using subtests from the Wechsler Intelligence Scale for Children, Fifth Edition (WISC-V)^33^ and the Clinical Evaluation of Language Fundamentals, Fourth Edition (CELF-4).^34^ The WISC-V provides five primary index scores, and these are combined to provide the full-scale intelligence quotient (IQ). The scaled scores for the core subtests of the Verbal Comprehension Index, the Vocabulary and Similarities, and the scaled subtest from CELF-4 on Recalling Sentences were used.

### MRI acquisition

The MRIs at age 10 years of age were performed using the General Electric 3.0-T MRI system (GE Healthcare, Milwaukee, WI) without sedation. The protocol included 3D T1-weighted images with a BRAVO SPRGR sequence of 400 milliseconds, a field of view of 240 × 240 mm^2^, a flip angle of 12°, a voxel size of 1 × 0.938 × 0.938 mm^3,^ and a slice thickness of 1.0 mm, as previously described.^31^ The scans were assessed by a neuroradiologist.

### Atlas-based segmentation and brain volumes

**Fig. 2:**
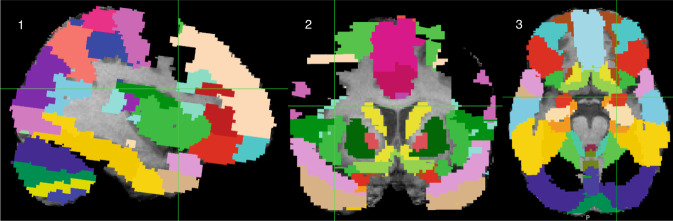
Automated anatomical labeling (AAL) atlas registered to one individual T1-weighted image. Sagittal view of the registered AAL atlas. 2 Coronal view of the registered AAL atlas. 3. Axial view of the registered AAL atlas.

The pre-processing steps of the T1-weighted 3D MRI images comprised of reorientation, removal of non-brain tissue, and neck-cropping.^31,35,36^ The pre-processed brain images were segmented into 45 anatomical regions per hemisphere, using the automated anatomical labeling (AAL) atlas.^37^ This was co-registered to the native space of each child using affine registration with the FLIRT linear image registration tool (FMRIB, Oxford, UK), as shown in Fig. 2.^38,39^ A visual inspection was performed for each child at each step. The cm^3^ volumes of the regions were determined by using a script written in MATLAB (MathWorks, MA).

### Brain asymmetry

Analyses of asymmetry were performed using the formula AI = (VL – VR)/(VL + VR)*100, where V was volume, L was the left side, and R was the right side. The AIs varied between −100 and +100. Positive values indicated left-sided preponderance and values near to zero indicated bilateral lateralization.^40^

### Cortical thickness analysis

The cortical thickness was analyzed using FreeSurfer Version 7.2.0 (Harvard University, MA)^41^ on the Linux operating system (The Linux Foundation, CA). The automated FreeSurfer pipeline for cortical thickness calculation has been previously described.^42,43^ In brief, the analysis pipeline consists of processing T1 images with intensity nonuniformity correction, skull stripping, affine transformation to the Montreal Neurological Institute (MNI) template, and linear and non-linear normalization to a standard spherical brain atlas, to match the cortical geometry across individuals.

### Language-related regions of interest

Language-related regions were pre-defined and selected based on previous studies.^5–7^

We included the canonical language regions described as Broca’s area, namely the triangular and opercular part of the inferior frontal gyri, and Wernicke’s area, within the superior temporal gyri.^4^ We also included the middle and inferior temporal gyri, the inferior parietal gyri, namely angular and supramarginal gyri, Heschl’s gyrus, and the supplementary motor area.

### Statistical analyses

The data were tested for normality and homogeneity. Unadjusted analyses for both groups were compared with the Student’s *t*-test for normally distributed continuous variables and the Mann–Whitney *U*-test for non-normally distributed continuous variables. The categorical variables were compared using Pearson’s chi-square, linear-by-linear association, and Fisher’s exact test as appropriate.

The brain volumes, AIs, cortical thickness, and scaled scores from the language assessments from both groups were compared using linear regression, fitted using generalized estimating equation (GEE) with robust estimation of standard errors to allow for any correlations among multiple births within a family. Separate models for each pre-defined brain volume region, AI, cortical thickness, or language subtest were used as the dependent variable, and group status (EPT/term-born) as a predictor. The analyses of brain volumes and AIs were adjusted for sex, and age at MRI and ICV. Cortical thickness analyses were adjusted for sex and age at MRI. Cortical thickness does not scale with ICV and it is not recommended as a covariate for this variable.^15^ The language assessments were adjusted for sex and maternal education and then repeatedly adjusted for home language.

Partial correlations, adjusted for sex, age at MRI, and maternal education, were performed. First, we correlated the three scaled language assessments (the vocabulary subtest, similarities subtest, and recalling sentences subtest) and the summed volume of the language-related regions and the AI of the summed language-related regions. Second, we correlated the scaled language assessments with the canonical language regions for cortical thickness, namely the opercular and triangular inferior frontal gyrus and the superior temporal gyrus.

To ameliorate the investigation of EPT birth per se the partial correlations were repeated without the four children born EPT with ASD. Analyses were then repeated adding home language and then full-scale IQ as covariates.

Outliers were detected graphically using boxplot diagrams and scatter plots and statistical analyses were repeated without the outliers. The results remained similar and all the children were kept in the final analyses.

Statistical analyses were performed using SPSS, version 28 and the statistical significance level was *p* < 0.05.

The Benjamini–Hochberg procedure^44^ was used to account for multiple comparisons, which is a commonly used method in studies with a similar design.^5,45,46^ The original *p*-values were compared to the critical value, defined as i/m*Q, where *i* *=* the rank, *m* = the number of tests and *Q* *=* the false-discovery rate (FDR), which was set to 0.05.^44^ The number of tests (*m*) for the analyses were the number of brain volumes, the number of cortical thickness regions analyzed or the number of language tests.

## Results

### Study population

Out of the 128 surviving children born EPT during the study period, there were 50 children included born EPT that had high-quality MRI data and out of these 41 children had developmental assessments (Fig. 1). We approached the families of 77 potential controls born at term, 37 had high-quality MRI data available and in addition 29 controls had developmental assessments, Fig. 1.

The characteristics of the two groups are summarized in Table 1. We found no significant differences in age at the time of MRI, age at language assessments, sex, the mother’s educational level, or handedness. However, a higher proportion of children used other languages than Swedish in the household in the EPT group. The perinatal and clinical characteristics of the EPT-born children are listed in Supplementary Tables 1 and 2. The drop-out analyses of the 50 children born EPT with MRI data showed that the non-participants had lower GAs and higher rates of sepsis and bronchopulmonary dysplasia, (Supplementary Table 1). Supplementary Table 2 compares the children born EPT, with and without both MRI and language assessments available, and the groups did not differ. Four children with MRI data and language assessments had ASD (Supplementary Table 2).

**Table 1:** Comparisons of characteristics and assessment scores of children born EPT and at term-age who underwent MRI scans at 10 years of age and language assessments and full-scale IQ tests at 12 years of age.

	Children born EPT	Term-born controls	Mean difference (95% CI)	*p*-value
MRI scans at 10 years of age, number	50	37		
Gestational age, median (range) weeks	25.6 (23.6–26.6)	40.0 (37.3–41.6)	-	^a^ < 0.001
Birth weight, mean (SD), g	848 (149)	3735 (459)	-	^b^ < 0.001
Age at scan, median (range) years	10.3 (9.0–11.4)	10.2 (8.3–11.6)	-	^a^0.63
Sex male, n (%)	23 (46)	18 (49)	-	^c^0.81
Intracranial volume, mean (SD), cm^3^	1390 (114)	1440 (110)	-	^b^0.042
Righthanded, n (%)	42 (84)	35 (95)	-	^c^0.25
Multiple births, n (%)	8 (16)	0 (0)	-	-
Language assessments at 12 years of age, number	41/50	29/37		
Age at developmental follow-up, median (range) years	12.2 (11.8–12.8)	12.1 (11.6–12.8)	-	^a^0.40
Gestational age, median (range) weeks	25.6 (23.6–26.6)	40.0 (38.0–41.6)	-	^a^ < 0.001
Birth weight, mean (SD), g	841 (153)	3809 (432)	-	^b^ < 0.001
Age at scan, median (range) years	10.5 (9.1–11.4)	10.3 (9.0–11.6)	-	^a^0.67
Sex male, n (%)	20 (49)	13 (45)	-	^c^0.74
Intracranial volume, mean (SD), cm^3^	1386 (118)	1453 (103)	-	^b^0.018
Righthanded, n (%)	33 (80)	27 (93)	-	^c^0.27
Multiple births, n (%)	6 (15)	0 (0)	-	-
Mothers with university-level education, n (%)	26 (63)	18 (62)	-	^c^0.91
Everyone in the household spoke Swedish, n (%)	28/38 (74)	26/27 (96)	-	^d^0.020
Vocabulary subtest (WISC-V), mean (SD) scores	10.4 (3.9)	13.0 (3.6)	–2.8 (–4.4, –0.7)	^b^0.007 ^e^0.004
Similarities subtest (WISC-V), mean (SD) scores	10.4 (3.0)	13.3 (2.7)	–2.9 (–4.3, –1.6)	^b^ < 0.001 ^e^ < 0.001
Full-scale IQ, mean (SD) scores	96.2 (14.6)	110.1 (12.3)	–14.6 (–21.3, –8.0)	^b^ < 0.001 ^e^ < 0.001
CELF-4, Recalling sentences, mean (SD) scores	8.6 (2.7)	10.1 (2.6)	–1.5 (–2.8, –0.2)	^b^0.013 ^e^0.018

^a^Mann–Whitney *U*-test.

^b^Student’s *t*-test.

^c^Pearson chi-square.

^d^Fisher’s exact test.

^e^Generalized estimating equations adjusted for sex and maternal education.

### Language assessments at 12 years

Both the unadjusted analyses and the analyses that were adjusted for sex and maternal education demonstrated significantly lower language scores for the children born EPT, Table 1. The results did not change when analyses were repeated adding home language as a covariate.

### Brain volumes

The children born EPT had smaller adjusted brain volumes for all the language-related regions than the term-born controls, except for Heschl’s gyrus on the left side (Table 2). The unadjusted brain volumes are in Supplementary Table 3a) and these show reduced brain volumes for all language-related regions. The adjusted mean difference for the summed language regions was −3.9 cm^3^ (95% CI −6.0 to −1.9) and the unadjusted mean difference was −12.0 cm^3^ (CI −20.0 to −4.1) between the children born EPT and the term-born controls.

**Table 2:** Comparison of brain volumes for language-related regions between children born EPT and term-born controls using generalized estimating equations.

Brain region	Children born EPT (*n* = 50)	Term-born controls (*n* = 37)	Mean difference, (95% CI), adjusted	*p*-value adjusted
Supplementary motor area right, mean (SD) cm^3^	14.97 (0.22)	15.23 (0.35)	–0.26 (−0.39, −0.13)	**<0.001**
Supplementary motor area left, mean (SD) cm^3^	13.56 (0.21)	13.80 (0.33)	−0.24 (−0.36, −0.11)	**<0.001**
Inferior frontal gyrus, triangular right, mean (SD) cm^3^	13.60 (0.21)	13.84 (0.40)	−0.24 (−0.37, −0.10)	**<0.001**
Inferior frontal gyrus triangular left, mean (SD) cm^3^	15.83 (0.23)	16.14 (0.48)	−0.31 (−0.47, −0.14)	**<0.001**
Inferior frontal gyrus opercular right, mean (SD) cm^3^	9.03 (0.14)	9.16 (0.23)	−0.13 (−0.21, −0.046)	**0.002**
Inferior frontal gyrus opercular left, mean (SD) cm^3^	6.37 (0.11)	6.46 (0.13)	−0.094 (−0.15, −0.041)	**<0.001**
Heschl’s gyrus right, mean (SD) cm^3^	1.53 (0.028)	1.55 (0.050)	−0.025 (−0.042, −0.008)	**0.004**
Heschl’s gyrus left, mean (SD) cm^3^	1.36 (0.028)	1.38 (0.043)	−0.016 (−0.033, −0.001)	0.069
Angular gyrus right, mean (SD) cm^3^	11.45 (0.19)	11.64 (0.25)	−0.19 (−0.29, −0.089)	**<0.001**
Angular gyrus left, mean (SD) cm^3^	6.98 (0.12)	7.09 (0.15)	−0.12 (−0.18, −0.057)	**<0.001**
Supramarginal gyrus right, mean (SD) cm^3^	12.26 (0.18)	12.46 (0.33)	−0.20 (−0.30, −0.089)	**<0.001**
Supramarginal gyrus left, mean (SD) cm^3^	7.19 (0.11)	7.31 (0.14)	−0.12 (−0.17, −0.058)	**<0.001**
Superior temporal gyrus right, mean (SD) cm^3^	19.61 (0.31)	19.94 (0.44)	−0.33 (−0.50, −0.16)	**<0.001**
Superior temporal gyrus left, mean (SD) cm^3^	13.61 (0.22)	13.81 (0.31)	−0.20 (−0.32, −0.078)	**0.001**
Middle temporal gyrus right, mean (SD) cm^3^	27.46 (0.41)	27.87 (0.59)	−0.41 (−0.64, −0.18)	**<0.001**
Middle temporal gyrus left, mean (SD) cm^3^	28.93 (0.41)	29.40 (0.60)	−0.47 (−0.71, −0.24)	**<0.001**
Inferior temporal gyrus right, mean (SD) cm^3^	22.03 (0.33)	22.33 (0.47)	−0.31 (−0.49, −0.12)	**0.001**
Inferior temporal gyrus left, mean (SD) cm^3^	18.40 (0.26)	18.69 (0.40)	−0.29 (−0.45, −0.14)	**<0.001**
Summed language regions, mean (SD) cm^3^	244.17 (3.43)	248.11 (5.23)	−3.94 (−5.96, −1.92)	**<0.001**

Results adjusted for sex, intracranial volume, and age at MRI.

Bold values are significant at *p* < 0.05. All significant results remained after the Benjamini–Hochberg procedure.

### Brain asymmetry

No significant differences were found for the AIs between the children born EPT and the term-born controls after adjustments for sex, age at MRI, and ICV (Table 3). A leftward volumetric asymmetry was detected in both groups for the inferior frontal gyrus, the triangular part, and the middle temporal gyrus. The other pre-defined regions demonstrated rightward volumetric asymmetry. The same pattern was found in the unadjusted analyses presented in Supplementary Table 3b).

**Table 3:** Comparison of asymmetry indexes for language-related regions between children born EPT and term-born controls using generalized estimating equations.

Brain region asymmetry	Children born EPT (*n* = 50)	Term-born controls (*n* = 37)	Mean difference, (95% CI), adjusted	*p*-value adjusted	Side dominance
Supplementary motor area, mean (SD) AI	–4.93 (0.45)	–4.93 (0.42)	–0.004 (–0.19, 0.18)	0.97	Right
Inferior frontal gyrus, triangular, mean (SD) AI	7.62 (0.30)	7.68 (0.27)	–0.055 (–0.18, 0.071)	0.37	Left
Inferior frontal gyrus, opercular, mean (SD) AI	–17.33 (0.56)	–17.28 (0.40)	–0.046 (–0.24, 0.15)	0.65	Right
Heschl’s gyrus, mean (SD) AI	–5.82 (1.10)	–6.08 (1.30)	0.26 (–0.29, 0.80)	0.35	Right
Angular gyrus, mean (SD) AI	–24.44 (0.35)	–24.31 (0.49)	–0.020 (–0.19, 0.15)	0.82	Right
Supramarginal gyrus, mean (SD) AI	–26.06 (0.49)	–26.05(0.90)	–0.010 (–0.21, 0.19)	0.93	Right
Superior temporal gyrus, mean (SD) AI	–18.06 (0.32)	–18.16 (0.55)	–0.11 (–0.93, 0.31)	0.29	Right
Middle temporal gyrus, mean (SD) AI	2.63 (0.19)	2.67 (0.30)	–0.046 (–0.16, 0.07)	0.42	Left
Inferior temporal gyrus, mean (SD) AI	–8.98 (0.25)	–8.89 (0.31)	–0.82 (–0.20, 0.042)	0.19	Right
Summed language regions, mean (SD) AI	–8.07 (0.091)	–8.05 (0.10)	–0.21(–0.061, 0.022)	0.32	Right

Results adjusted for sex, intracranial volume, and age at MRI.

### Cortical thickness

As demonstrated in Table 4, most of the language-related regions demonstrated reduced thickness for the children born EPT, compared to the term-born controls, when adjusted for sex and age at MRI. However, there were no significant differences between the cortical thickness for the inferior frontal gyrus, triangular and opercular region on the left side, or the inferior temporal gyrus on the right side. The unadjusted comparisons in Supplementary Table 3c) were in line with the adjusted results.

**Table 4:** Comparison of cortical thickness for language-related regions between children born EPT and term-born controls using generalized estimating equations.

Cortical thickness, brain region	Children born EPT (*n* = 50)	Term-born controls (*n* = 37)	Mean difference, (confidence interval), adjusted	*p*-value adjusted
Inferior frontal gyrus, triangular right, mean (SD) cm^3^	2.70 (0.14)	2.79 (0.12)	−0.085 (–0.15, −0.020)	**0.011**
Inferior frontal gyrus triangular left, mean (SD) cm^3^	2.83 (0.13)	2.88 (0.12)	−0.051 (–0.11, 0.004)	0.071
Inferior frontal gyrus opercular right, mean (SD) cm^3^	2.79 (0.13)	2.92 (0.11)	−0.13 (−0.18, −0.08)	**<0.001**
Inferior frontal gyrus opercular left, mean (SD) cm^3^	2.90 (0.12)	2.93 (0.12)	−0.038 (0.15, −0.092)	0.16
Inferior parietal gyrus right, mean (SD) cm^3^	2.76 (0.13)	2.86 (0.25)	−0.10 (−0.16, −0.051)	**<0.001**
Inferior parietal gyrus left, mean (SD) cm^3^	2.63 (0.12)	2.79 (0.12)	−0.16 (−0.21, −0.11)	**<0.001**
Superior temporal gyrus right, mean (SD) cm^3^	3.01 (0.16)	3.13 (0.11)	−0.12 (−0.18, −0.07)	**<0.001**
Superior temporal gyrus left, mean (SD) cm^3^	3.04 (0.17)	3.12 (0.13)	−0.080 (−0.14, −0.016)	**0.014**
Middle temporal gyrus right, mean (SD) cm^3^	2.92 (0.17)	3.08 (0.16)	−0.16 (−0.23, −0.086)	**<0.001**
Middle temporal gyrus left, mean (SD) cm^3^	2.88 (0.16)	3.16 (0.17)	−0.29 (−0.34, −0.21)	**<0.001**
Inferior temporal gyrus right, mean (SD) cm^3^	3.09 (0.16)	3.07 (0.13)	0.016 (−0.047, 0.079)	0.62
Inferior temporal gyrus left, mean (SD) cm^3^	3.02 (0.18)	3.11 (0.16)	−0.093 (−0.17, −0.021)	**0.012**

Results adjusted for sex and age at MRI.

Bold values are significant at *p* < 0.05. All significant results remained after the Benjamini–Hochberg procedure.

### MRI assessments and association with language outcomes

Associations between language outcomes at 12 years of age and MRI assessments are presented in Table 5 and the correlations were adjusted for sex, age at MRI, and maternal education. In the EPT group, the scaled scores for the vocabulary subtest of the WISC-V (*r* = 0.37, *p* = 0.021) and recalling sentences of the CELF-4 (*r* = 0.37, *p* = 0.028) were positively associated with the volume of the summed language regions. These results remained similar after adjusting for home language, Supplementary Table 4. There was no association between the volume of the summed language regions and language outcomes for term-born controls (Table 5).

**Table 5:** Partial correlations between language outcomes and MRI assessments.

	Vocabulary subtest	Similarities subtest	Recalling sentences
	*r* (*p*-value)	*r* (*p*-value)	*r* (*p*-value)
	Children born extremely preterm *n* = 41		
Volume of summed language regions	**0.37 (0.021)**	0.18 (0.29)	**0.37 (0.028)**
Asymmetry index for the summed language regions	0.14 (0.41)	–0.16 (0.33)	–0.001 (0.99)
Cortical thickness of the superior temporal gyrus	–0.090 (0.59)	–0.14 (0.41)	0.090 (0.60)
Cortical thickness of the inferior frontal gyrus, triangular	0.14 (0.42)	–0.042 (0.81)	–0.020 (0.24)
Cortical thickness of the inferior frontal gyrus, opercular	–0.30 (0.067)	–0.12 (0.47)	–0.12 (0.40)

	Term-born controls *n* = 29		
Volume of summed language regions	–0.056 (0.79)	–0.051 (0.81)	0.22 (0.28)
Asymmetry Index for the summed language regions	0.14 (0.51)	0.12 (0.55)	0.14 (0.48)
Cortical thickness of the superior temporal gyrus	–0.21 (0.18)	–0.006 (0.98)	–0.067 (0.75)
Cortical thickness of the inferior frontal gyrus, triangular	0.022 (0.91)	–0.035 (0.87)	–0.040 (0.85)
Cortical thickness of the inferior frontal gyrus, opercular	–0.24 (0.23)	–0.30 (0.13)	**–0.47 (0.015)**

Correlations were adjusted for sex, age at MRI, and maternal education.

Bold values are significant at *p* < 0.05. All significant results remained after the Benjamini–Hochberg procedure.

The volumetric AI for the summed language regions was not associated with language outcomes in either group (Table 5).

There were no significant associations between language outcomes and cortical thickness for children born EPT (Table 5).

The cortical thickness of the inferior frontal gyrus and opercular part was negatively associated with the scaled scores of recalling sentences of CELF-4 (*r* = -0.47 *p* = 0.015) for term-born controls (Table 5).

The analyses for children born EPT were repeated after the exclusion of the four children with ASD and the results remained similar, (Supplementary Table 5).

Brain volumes and cortical thickness did not remain significantly associated with language outcomes after full-scale IQ was added as a covariate (Supplementary Table 6).

Results that were significant at *p* < 0.05 remained significant after the Benjamini–Hochberg procedure was applied.

## Discussion

This study demonstrates that the volume and cortical thickness of pre-defined language-related regions were generally reduced in children born EPT when they were compared to term-born controls at 10 years of age. There was no difference in volumetric asymmetry in the language-related regions between the two groups. Children born EPT had lower language assessment scores than term-born controls at 12 years of age. We found a positive association between the volume of the summed language regions and the language outcomes in children born EPT. Cortical thickness was not related to language outcomes in children born EPT. There was a negative association between one of the three studied language outcomes, namely recalling sentences, and cortical thickness within the inferior frontal gyrus in the term-born controls. The significant associations between brain volume or cortical thickness with language outcomes did not remain significant after the data were adjusted for full-scale IQ. We found no associations between volumetric brain asymmetry and language outcomes.

The volumes of language-related regions were reduced in children born EPT, even after correcting for ICV. Previous studies have investigated global brain volumes after EPT birth at this age and the results showed that the reduced ICV was mainly driven by a reduction in WM. We already knew that brain growth tends to be generally stunted after EPT birth, but the current study demonstrates that the language regions were more affected and possibly particularly vulnerable.^31^

The third trimester is a critical window for brain growth.^47^ Children born EPT spend this time in neonatal intensive care units (NICU) with altered sensory inputs, rather than in the protected in-utero environment.^47^ Children born EPT are exposed to more electronic sounds and noise in these units and more than 150 hours less parental spoken language over 12 weeks compared to in an in-utero environment.^48^ It is possible that these experiences may alter their fine-tuned neural processes.^48^

Other studies have investigated very and extremely preterm children at the whole brain level, using voxel-based morphometry.^22,49–51^ These reported that the temporal regions, and the inferior frontal gyri, were particularly susceptible to volumetric reductions for children born very preterm during adolescence^49,50^ and into adulthood.^51^ Fewer studies have focused on children born EPT, but we previously found that areas within the temporal lobes were reduced at term age and that this pattern persisted into childhood when examined with voxel-based morphometry, which suggests that these findings are consistent over time.^22,23^

Our study found positive correlations between language outcomes and the volume of the summed language regions in children born EPT, prior to adjustment for full-scale IQ. These positive associations remained after the exclusion of children born EPT with ASD. We are not aware of any previous studies that investigated associations between the volume of language-related regions and language outcomes when children born EPT reached school age. These results were in line with a previous study of very preterm adolescents that reported that frontal and temporal regions were associated with language performance.^50^

There were no significant correlations between the summed brain volume of language areas and language outcomes for the term-born controls. One possible explanation could be that the language regions were more integrated in term-born controls than for the children born EPT. When children are born EPT the neural activity is altered compared to term-born controls.^36^ EPT children have been shown to have reduced intrinsic ignition, which is a reduced ability for local activity to promote global activity.^36^

We included results adjusted for full-scale IQ, but we are aware that this decreased variations in the data because language performance was incorporated into the IQ calculation.^52,53^ Whether or not to adjust for full-scale IQ in neurodevelopmental studies has been previously discussed and it has been suggested not to use full-scale IQ as a covariate since it cannot be separated from the effect of the studied condition.^52,53^ However, a review article about children born preterm and language development suggested adding results with cognitive ability as a control variable to decipher whether language difficulties are linked to overall cognitive abilities, and we agree that it adds important information.^1^ There were no significant associations between the summed brain volume of language-related regions and language performance in the children born EPT after correction for full-scale IQ. This result was expected, because children born with EPT are known to have developmental problems and language is one of the affected domains.^52^ We also know from previous studies that associations between neurodevelopmental outcomes and brain volumes are often weakened or disappear after adjustments for full-scale IQ.^54^ It is however essential that specific language assessments are carried out alongside general assessments of cognition and motor functions when children are followed after EPT birth because these functions are interconnected.

There were no differences in volumetric asymmetry between the two groups in any of the regions examined and most regions were right-lateralized. Brain asymmetry was not related to any language assessments, as reported by a previous study of very preterm children.^55^ However, functional MRI studies have shown reduced asymmetry for children born preterm, compared to term-born controls, and that left-sided lateralization was related to positive outcomes for very preterm children. This suggests that asymmetry may be more predictive of language outcomes when it is analyzed with functional MRI methods.^56^ The lack of differences in volumetric asymmetry between children born EPT and term-born controls and the lack of association with outcome could also be due to the strong genetic influence underlying brain asymmetry, as previously suggested.^55^ Our group has before related brain asymmetry at term age to language outcomes in a partly overlapping cohort and found that asymmetry scores were related to language outcomes for children born EPT with ASD, but not for those born EPT without ASD.^5^ In the current study, the non-significant correlations between asymmetry and language outcomes remained when children born EPT with ASD were excluded, in line with what has previously been reported at term age.^5^ The number of children born EPT with ASD was too small to investigate associations. However, this could be an area for future research.

Children born EPT had reduced cortical thickness in language-related regions when they were compared with the term-born controls. This result was in line with most of the evidence to date on children born very preterm or with a very low birth weight.^17,18^

The cortical thickness of the regions examined was not associated with language outcomes for children born EPT. Previous literature has indicated that cortical thickness was not associated with cognitive outcomes.^18,57^ However, positive relationships have been reported between cortical thickness and language outcomes for children born EPT and very preterm in childhood.^16,58^ Cortical thickness is age-dependent and these differences could be due to the age at examination, the used covariates, and different imaging softwares.^15^ Even if the children born EPT in the current study had significantly reduced language scores compared with the term-born children they performed relatively well. It has been previously demonstrated that children born preterm with more complications and perinatal injury have stronger relationships between cortical thickness and cognitive outcome, which could possibly contribute to the non-significant findings.^17^

We also found a significant negative relationship in the term-born group when it came to recalling sentences and cortical thickness of the inferior frontal gyri, opercular region. The rapid early growth of the cerebral cortex slows down as cortical thinning begins at around 7 years of age because of myelination, pruning, and optimization of the nervous system.^16^ This negative relationship could have been due to a developed pruning mechanism. Negative associations between cortical thickness and intelligence have been shown in healthy populations from 10 years of age.^59^

### Strengths and limitations

This study had several limitations. The sample size was relatively small which may have prevented statistical differences from being discerned, especially weaker associations. However, the study should have the power to detect moderate to strong associations. We included all available children born EPT during the pre-defined time period in our region and the sample size is on par with previous similar MRI studies.^16,60^ The results, particularly the associations between neuroimaging data and language outcomes, should be confirmed in larger cohorts.

The group characteristics demonstrated a higher proportion of university-level education and a lower percent of non-Swedish present in the household for both children born EPT and term-born controls than in the general population in Sweden.^61,62^

Another limitation was that we did have some drop-out between the MRI scans at 10 years of age and the developmental assessments at 12 years of age.

The drop-out analyses demonstrated that the non-participants without MRI data had a higher rate of sepsis and BPD, and enrollment could have altered the results. We did however not see these differences in the drop-out analysis between participants with complete MRI data and language assessment data and those without complete data.

### Conclusion

This study found that children born with EPT demonstrated reduced brain volumes and cortical thickness in their language-related regions by 10 years of age and that the effect was equal for both the left and right hemispheres. Our findings suggest that the volume of language-related regions could be potential biomarkers for language impairment in children born EPT. In contrast, cortical thickness and structural asymmetry of language-related regions were not associated with language performance. We recommend future research on the effects of early language interventions on brain morphology. It would also be useful to further study asymmetry using other MRI methods, such as functional MRI because previous research indicates that this may predict outcomes.

### Supplementary information

Supplementary Information

## Data Availability

Full datasets generated during and/or analyzed during the current study are available from the corresponding author upon reasonable request.
