# Supplementary material for: Language performance and brain volumes, asymmetry, and cortical thickness in children born extremely preterm
Source: Pediatr Res. 2023 Nov 3;95(4):1070–9. doi: 10.1038/s41390-023-02871-0 (PMC10920199; doi:10.1038/s41390-023-02871-0)
Supplement: Supplementary file 1 — Supplementary Information [file 41390_2023_2871_MOESM1_ESM.pdf]

Supplementary material

	Included children born EPT with MRI (n=50)	Children born EPT who declined/ had low-quality MRI (n=42)	*p*-value
Gestational age, median (range) weeks	25.6 (23.6–26.6)	25.1 (23.3–26.6)	[b]0.040
Birth weight, mean (SD), g	848 (149)	790 (170)	[a]0.086
Sex male, n (%)	23 (46)	26 (62)	[c]0.13
Mothers with university level education, n (%)	27/43 (63)	13/18 (72)	[b]0.48
Handedness, righthanded, n (%)	42 (84)	n=31, 23 (74)	[c]0.32
Everyone in household spoke Swedish, n (%)	28/38 (74)	14/18 (78)	[d]1.0
Clinical Autism diagnosis at the time of MRI, n (%)	6 (12)	6 (14)	[c]0.75
Multiple births, n (%)	8 (16)	8 (19)	[c]0.70
Antenatal steroids, n (%)	47 (94)	39 (93)	[d]1.0
Postnatal steroids, n (%)	5 (10)	10 (23)	[c]0.074
Patent ductus arteriosus, n (%)	35 (70)	32 (76)	[c]0.51
Patent ductus arteriosus ligation, n (%)	16 (32)	14 (33)	[c]0.89
Patent ductus arteriosus medically treated, n (%)	33 (66)	31 (74)	[c]0.42
Sepsis, n (%)	35 (70)	38 (90)	[d]0.020
Intraventricular haemorrhage grade 1-2, n (%)	15 (30)	19 (45)	[c]0.13
Small for gestational age <2SD, n	4 (8)	5 (12)	[d]0.73
Necrotizing enterocolitis, n (%)	7 (14)	7 (17)	[c]0.94
Any retinopathy of prematurity, n (%)	38 (75)	33/38 (87)	[c]0.20
WM abnormalities, none, n (%)	26/48 (54)	17/38 (45)	[e]0.23
mild, n (%)	21/48 (44)	18/38 (37)	
moderate, n	1/48 (2)	3/38 (8)	
Bronchopulmonary dysplasia, n (%)	18 (36)	24 (57)	[c]0.023

Supplementary Table 1. Drop-out analyses for children born EPT with MRI data.
[a]Student's t test, [b]Mann-Whitney U test, [c]Pearson chi-square, [d]Fisher's exact test, [e]Linear-by-linear association. MRI = magnetic resonance imaging. WM = white matter

	Included children born EPT with MRI and language outcome data (n=41)	Children born EPT who declined/ had low-quality MRI or no data for language outcomes (n=51)	*p*-value
Gestational age, median (range) weeks	25.6 (23.6–26.6)	25.4 (23.3–26.6)	[b]0.13
Birth weight, mean (SD), g	842 (153)	806 (167)	[a]0.29
Sex male, n (%)	20 (49)	29 (57)	[c]0.44
Maternal education, dichotomized, university level, n (%)	26/41 (63)	14/20 (70)	[c]0.66
Handedness, righthanded, n (%)	33 (80)	32/40 (80)	[e]0.67
Everyone in household spoke Swedish, n (%)	28/38 (74)	14/18 (78)	[d]1.0
Clinical autism diagnosis at the time of MRI, n (%)	4 (10)	8 (16)	[d]0.54
Multiple births, n (%)	6 (16)	10 (19)	[c]0.53
Antenatal steroids, n (%)	38 (93)	48 (94)	[d]1.0
Postnatal steroids, n (%)	4 (10)	11 (21)	[d]0.11
Patent ductus arteriosus, n (%)	28 (68)	39 (76)	[c]0.38
Patent ductus arteriosus ligation, n (%)	14 (34)	16 (31)	[c]0.78
Patent ductus arteriosus medically treated, n (%)	26 (66)	38 (74)	[c]0.25
Sepsis, n (%)	29 (71)	44 (86)	[d]0.067
Intraventricular haemorrhage grade 1-2, n (%)	14 (34)	20 (39)	[c]0.61
Small for gestational age <2SD, n	4 (10)	5 (10)	[d]0.99
Necrotizing enterocolitis, n (%)	7 (17)	7 (15)	[c]0.66
Any retinopathy of prematurity, n (%)	33/41 (80)	33/48 (69)	[c]0.45
WM abnormalities, none, n (%)	24/39 (52)	19/47 (40)	[e]0.051
mild, n (%)	14/39 (42)	25/47 (43)	
moderate, n	1/39 (2)	3/47 (7)	
Bronchopulmonary dysplasia, n (%)	17 (41)	25 (49)	[c]0.37

Supplementary Table 2. Drop-out analyses for children born EPT with MRI and language outcome data.

[a]Student's t test, [b]Mann-Whitney U test, [c]Pearson chi-square, [d]Fisher's exact test, [e]Linear-by-linear association. MRI= magnetic resonance imaging, WM = white matter.

Brain region volume	Children born EPT (n=50)	Term-born controls (n=37)	Mean difference, (95% CI)	*p*-value
Supplementary motor area, right, mean (SD) cm^3	14.8 (1.2)	15.5 (1.1)	–0.76 (–1.3, –0.27)	**[a]0.003**
Supplementary motor area, left, mean (SD) cm^3	13.4 (1.1)	14.1 (1.0)	–0.70 (–1.1, –0.24)	**[a]0.003**
Inferior frontal gyrus, triangular, right, mean (SD) cm^3	13.4 (1.1)	14.1 (1.1)	–0.71 (–1.2, –0.24)	**[a]0.004**
Inferior frontal gyrus, triangular, left, mean (SD) cm^3	15.6 (1.2)	16.5 (1.3)	–0.85 (–1.4, –0.30)	**[a]0.003**
Inferior frontal gyrus, opercular, right, mean (SD) cm^3	8.9 (0.7)	9.3 (0.7)	–0.44 (–0.74, –0.13)	**[a]0.006**
Inferior frontal gyrus, opercular, left, mean (SD) cm^3	6.3 (0.5)	6.6 (0.4)	–0.30 (-0.51, –0.10)	**[a]0.005**
Heschl's gyrus, right, mean (SD) cm^3	1.5 (0.1)	1.6 (0.1)	–0.073 (-0.12, –0.024)	**[a]0.004**
Heschl's gyrus, left, mean (SD) cm^3	1.3 (0.1)	1.4 (0.1)	–0.057 (–0.10, –0.013)	**[a]0.011**
Angular gyrus, right, mean (SD) cm^3	11.3 (0.9)	11.9 (0.8)	–0.57 (–0.94, –0.19)	**[a]0.003**
Angular gyrus, left, mean (SD) cm^3	6.9 (0.6)	7.2 (0.5)	–0.34 (–0.56, –0.12)	**[a]0.003**
Supramarginal gyrus, right, mean (SD) cm^3	12.1 (1.0)	12.7 (0.9)	–0.60 (–1.0, –0.20)	**[a]0.004**
Supramarginal gyrus, left, mean (SD) cm^3	7.1 (0.6)	7.4 (0.5)	–0.34 (–0.58, -0.12)	**[a]0.004**
Superior temporal gyrus, right, mean (SD) cm^3	19.3 (1.5)	20.3 (1.3)	–1.0 (–1.6, –0.33)	**[a]0.003**
Superior temporal gyrus, left, mean (SD) cm^3	13.4 (1.1)	14.1 (0.9)	–0.64 (–1.1, –0.21)	**[a]0.005**
Middle temporal gyrus, right, mean (SD) cm^3	27.1 (2.2)	28.4 (1.9)	–1.3 (–2.2, –0.43)	**[a]0.004**
Middle temporal gyrus, left, mean (SD) cm^3	28.5 (2.3)	29.9 (2.0)	–1.4 (–2.3, –0.48)	**[a]0.003**
Inferior temporal gyrus, right, mean (SD) cm^3	21.7 (1.8)	22.7 (1.5)	–1.0 (–1.7, –0.31)	**[a]0.005**
Inferior temporal gyrus, left, mean (SD) cm^3	18.1 (1.5)	19.0 (1.2)	–0.9 (–1.4, –0.30)	**[a]0.003**
Summed language regions, mean (SD) cm^3	240.7 (19.4)	252.7 (17.1)	–12.0 (–20.0, –4.1)	**[a]0.003**

Supplementary Table 3. a) Unadjusted comparisons of brain volumes for language-related regions between children born EPT and term-born controls using Student's t-test.

Bold values are significant at $p<0.05$. All significant results remained after the Benjamini-Hochberg procedure.

Brain region asymmetry	Children born EPT	Term-born controls	*p*-value	Side dominance
Supplementary motor area, median (range) AI	–4.9 (–6.9, –3.6)	–4.9 (–5.9, –2.8)	0.73	Right
Inferior frontal gyrus, triangular, median (range) AI	7.7 (6.9–8.1)	7.7 (7.0–8.3)	0.60	Left
Inferior frontal gyrus, opercular, median (range) AI	–17.33 (–18.1, –14.5)	–17.28 (–18.9, –16.3)	0.66	Right
Heschl's gyrus, median (range) AI	–5.7 (–9.3, –3.3)	–6.1 (–10.0, –3.2)	0.16	Right
Angular gyrus, median (range) AI	–24.4 (–25.5, –23.0)	–24.3 (–26.6, –23.6)	0.74	Right
Supramarginal gyrus, median (range) AI	–26.0 (–27.6, –24.2)	–26.1 (–27.7, –24.5)	0.47	Right
Superior temporal gyrus, median (range) AI	–18.1 (–19.0, –17.2)	–18.1 (–20.9, –17.4)	0.93	Right
Middle temporal gyrus, median (range) AI	2.6 (1.9–3.1)	2.6 (2.2–3.8)	0.98	Left
Inferior temporal gyrus, median (range) AI	–8.9 (–9.9, –8.6)	–8.9 (–9.7, –7.3)	0.19	Right
Summed language regions, median (range) AI	–8.1 (8.6, –7.8)	–8.1 (8.3, –7.8)	0.31	Right

Supplementary Table 3. b) Unadjusted comparisons of asymmetry indexes (AI) for language-related regions between children born EPT and term-born controls using Mann-Whitney U test.

Cortical thickness, brain region	Extremely preterm children (n=50)	Term-born controls (n=37)	Mean difference, (95% CI)	*p*-value
Inferior frontal gyrus, triangular right, mean (SD) cm^3	2.70 (0.18)	2.79 (0.15)	–0.081 (–0.15, –0.011)	**0.012**
Inferior frontal gyrus triangular left, mean (SD) cm^3	2.84 (0.14)	2.89 (0.13)	–0.049 (–0.11, 0.010)	0.053
Inferior frontal gyrus opercular right, mean (SD) cm^3	2.79 (0.14)	2.92 (0.12)	–0.13 (–0.18, –0.070)	**<0.001**
Inferior frontal gyrus opercular left, mean (SD) cm^3	2.90 (0.13)	2.93 (0.13)	–0.035 (–0.020, 0.090)	0.21
Inferior parietal gyrus right, mean (SD) cm^3	2.76 (0.13)	2.86 (0.12)	–0.10 (–0.16, –0.048)	**<0.001**
Inferior parietal gyrus left, mean (SD) cm^3	2.64 (0.12)	2.79 (0.12)	–0.16 (–0.21, –0.10)	**<0.001**
Superior temporal gyrus right, mean (SD) cm^3	3.01 (0.17)	3.13 (0.12)	–0.12 (–0.18, –0.052)	**<0.001**
Superior temporal gyrus left, mean (SD) cm^3	3.04 (0.17)	3.12 (0.14)	–0.078 (–0.15, –0.010)	**0.024**
Middle temporal gyrus right, mean (SD) cm^3	2.92 (0.18)	3.08 (0.16)	–0.15 (–0.23, –0.078)	**<0.001**
Middle temporal gyrus left, mean (SD) cm^3	2.89 (0.18)	3.16 (0.17)	–0.27 (–0.35, –0.20)	**<0.001**
Inferior temporal gyrus right, mean (SD) cm^3	3.09 (0.18)	3.07 (0.14)	0.021 (–0.048, 0.090)	0.55
Inferior temporal gyrus left, mean (SD) cm^3	3.02 (0.19)	3.11 (0.17)	–0.086 (–0.16, –0.007)	**0.033**

Supplementary Table 3. c) Unadjusted comparisons of cortical thickness for language-related regions between children born EPT and term-born controls using Student's t-test.

Bold values are significant at $p<0.05$. All significant results remained after the Benjamini-Hochberg procedure.

	Children born extremely preterm n=38/41		
	Vocabulary subtest<br>*r* (*p*-value)	Similarities subtest<br>*r* (*p*-value)	Recalling sentences<br>*r* (*p*-value)
Volume of summed language regions	**0.41 (0.017)**	0.27 (0.13)	**0.40 (0.023)**
Asymmetry index for the summed language regions	0.11 (0.53)	0.16 (0.36)	0.017 (0.93)
Cortical thickness of the superior temporal gyrus	–0.088 (0.62)	–0.010 (0.63)	0.070 (0.70)
Cortical thickness of the inferior frontal gyrus, triangular	0.14 (0.43)	–0.18 (0.32)	0.070 (0.70)
Cortical thickness of the inferior frontal gyrus, opercular	–0.31 (0.074)	–0.12 (0.51)	–0.11 (0.56)

Supplementary Table 4. Partial correlations between language outcomes and MRI assessments for children born EPT, adjusted for sex, age at scan, maternal education, and home language.
Home language had missing data for 3/41 children and the number is reduced to n=38. Bold values are significant at $p<0.05$. All significant results remained after the Benjamini-Hochberg procedure.

	Children born extremely preterm n=37/41		
	Vocabulary subtest<br>*r* (*p*-value)	Similarities subtest<br>*r* (*p*-value)	Recalling sentences<br>*r* (*p*-value)
Volume of summed language regions	**0.42 (0.014)**	0.27 (0.12)	**0.39 (0.023)**
Asymmetry index for the summed language regions	0.16 (0.37)	0.20 (0.25)	–0.016 (0.93)
Cortical thickness of the superior temporal gyrus	–0.11 (0.55)	–0.18 (0.31)	0.020 (0.92)
Cortical thickness of the inferior frontal gyrus, triangular	0.37 (0.84)	0.014 (0.94)	–0.12 (0.50)
Cortical thickness of the inferior frontal gyrus, opercular	–0.32 (0.064)	–0.097 (0.58)	–0.11 (0.54)

Supplementary Table 5. Partial correlations between language outcomes and MRI assessments for children born EPT without an autism diagnosis (n=37), adjusted for sex, age at scan and maternal education.
Bold values are significant at $p<0.05$. All significant results remained after the Benjamini-Hochberg procedure.

	Children born EPT n=41		
	Vocabulary subtest<br>r (*p*-value)	Similarities subtest<br>r (*p*-value)	Recalling sentences<br>r (*p*-value)
Volume of summed language regions	0.20 (0.23)	–0.15 (0.37)	0.25 (0.14)
Asymmetry index for the summed language regions	–0.10 (0.95)	–0.008 (0.97)	–0.084 (0.63)
Cortical thickness of the superior temporal gyrus	0.049 (0.77)	0.012 (0.94)	0.25 (0.15)
Cortical thickness of the inferior frontal gyrus, triangular	0.32 (0.058)	0.11 (0.53)	–0.12 (0.49)
Cortical thickness of the inferior frontal gyrus, opercular	–0.20 (0.23)	–0.094 (0.58)	–0.030 (0.87)

	Term-born controls n=29		
	Vocabulary subtest<br>r (*p*-value)	Similarities subtest<br>r (*p*-value)	Recalling sentences<br>r (*p*-value)
Volume of summed language regions	–0.33 (0.11)	–0.34 (0.097)	0.10 (0.62)
Asymmetry Index for the summed language regions	0.14 (0.51)	0.12 (0.58)	0.14 (0.51)
Cortical thickness of the superior temporal gyrus	–0.23 (0.26)	0.18 (0.40)	0.063 (0.76)
Cortical thickness of the inferior frontal gyrus, triangular	0.012 (0.96)	–0.077 (0.72)	–0.076 (0.72)
Cortical thickness of the inferior frontal gyrus, opercular	–0.025 (0.91)	–0.11 (0.60)	–0.38 (0.061)

Supplementary Table 6. Partial correlations between language outcomes and MRI assessments, adjusted for sex, age at MRI, maternal education, and full-scale IQ.
